# Feedback Integrators for Mechanical Systems with Holonomic Constraints

**DOI:** 10.3390/s22176487

**Published:** 2022-08-29

**Authors:** Dong Eui Chang, Matthew Perlmutter, Joris Vankerschaver

**Affiliations:** 1 School of Electrical Engineering, Korea Advanced Institute of Science and Technology, Daejeon 34141, Korea; 2 Department of Mathematics, Universidade Federal de Minas Gerais, Belo Horizonte 31270-901, Brazil; 3 Center for Biosystems and Biotech Data Science, Ghent University Global Campus, Incheon 21985, Korea; 4 Department of Applied Mathematics, Computer Science and Statistics, Ghent University, 9000 Ghent, Belgium

**Keywords:** feedback integrator, numerical integration, holonomic constraint, first integral

## Abstract

The feedback integrators method is improved, via the celebrated Dirac formula, to integrate the equations of motion for mechanical systems with holonomic constraints so as to produce numerical trajectories that remain in the constraint set and preserve the values of quantities, such as energy, that are theoretically known to be conserved. A feedback integrator is concretely implemented in conjunction with the first-order Euler scheme on the spherical pendulum system and its excellent performance is demonstrated in comparison with the RATTLE method, the Lie–Trotter splitting method, and the Strang splitting method.

## 1. Introduction

The method of feedback integrators was proposed in [1,2] to numerically integrate the equations of motion for dynamical systems in order to preserve their domain manifolds and first integrals. The method is summarized as follows. Suppose that there is a given invariant set Λ of a continuous-time dynamical system on a manifold *P*, where Λ can be the intersection of level sets of the first integrals of the system. Embed *P* into some Euclidean space, extend the system to the ambient Euclidean space, and modify it outside Λ to turn Λ into a local attractor of the resulting dynamical system in the ambient space. Then, trajectories originating from points in Λ generated by integration of the modified dynamics remain in Λ theoretically and near Λ numerically, irrespective of the choice of numerical integration schemes. It is rigorously shown [1,3,4] that the discrete-time dynamical system derived from any one-step numerical integrator with uniform step size *h* for the modified continuous-time system has an attractor $\Lambda_{h}$ that contains Λ in its interior and converges to Λ as h→0+. In this procedure, the set of equations of motion of the modified dynamical system is called a feedback integrator. Feedback integrators can be implemented by any usual integration scheme such as Euler, Runge–Kutta, or Matlab ode45 in one single global Cartesian coordinate system for the ambient Euclidean space and do not require projecting numerical trajectories to a certain set or solving algebraic equations during integration.

Here, we propose a way to apply feedback integrators to mechanical systems with holonomic constraints, which is not addressed in [1,2]. Consider a symplectic manifold *P*, a symplectic submanifold *S* of *P*, and a Hamiltonian function *H* on *P*, where it is often the case that $P=T^{*}R^{n}$ for some *n* and *S* is the cotangent bundle of a set of holonomic constraints in $R^{n}$. The Hamiltonian function *H* defines a Hamiltonian vector field $X_{H}$ on *P* and the restriction H|S of *H* to *S* defines a Hamiltonian vector field, denoted $X_{H|S}$, on *S*. In general, $X_{H}$ does not coincide with $X_{H|S}$ on *S*; so, we employ the celebrated Dirac formula to extend the vector field $X_{H|S}$ from *S* to *P*, such that the dynamical system extends from *S* to *P*. The manifold *S* is an invariant set of the extended dynamical system on *P*. Thus, we can apply the usual method of feedback integrators to the extended system on *P* such that *S* is numerically well-preserved.

This paper is organized as follows. The Dirac formula is first reviewed for the sake of completeness; then, the procedure for implementing feedback integrators for mechanical systems with holonomic constraints is presented. A simulation study is carried out on the spherical pendulum system, which is a mechanical system with a holonomic constraint, to demonstrate the excellent performance of feedback integrators in preserving the constraint set, the total energy, and the vertical component of the angular momentum vector, in comparison with the RATTLE method, the Lie–Trotter method, and the Strang method. Refer to [5] for more information on the three methods. We conclude with a small-scale simulation of the planar pendulum to show that the feedback method generally gives rise to integrators that are computationally more efficient as well.

### Related Work

The numerical integration of mechanical systems with holonomic constraints has been an area of active research interest over the past decades. In this section, we briefly compare our approach with existing methods from the literature. Holonomic constraints can be viewed as index-1 differential-algebraic equations (DAEs) [6,7]. These systems can be integrated by direct discretization or by reformulating the DAE as an equivalent set of ODEs to which a standard numerical integration scheme may be applied. The resulting integration scheme typically involves the solution of a nonlinear equation representing the constraint. Another approach starts from the Hamiltonian or variational nature of mechanical systems to come up with discretizations that preserve the symplectic structure and the constraint manifold [5,8,9,10]. Such discretizations, the RATTLE algorithm [11] and its generalizations [12,13,14] chiefly among them, typically exhibit superior long-term integration properties compared with standard, nonsymplectic integration algorithms, and have found wide application in the numerical integration of mechanical and control systems [15,16,17]. This approach has also been extended to the case of classical field theories with constraints [18,19], or to systems with dissipation [20]. What all these methods have in common is that they seek to enforce the constraint equations directly, which requires special-purpose integration algorithms. By contrast, the feedback integrator method described in this paper modifies the equations of motion directly, to approximately conserve the constraint equations and other integrals of motion. As we pointed out before, the advantage of this approach is that it allows for standard, off-the-shelf numerical integrators to be used, such as the Euler or Runge–Kutta method.

## 2. Main Results

We first review the Dirac formula [21,22], explain how to construct feedback integrators for mechanical systems with holonomic constraints, and design a feedback integrator for the planar and spherical pendulum systems to demonstrate its excellent integration performance and versatility in comparison with the RATTLE method, the Lie–Trotter method, and the Strang method.

### 2.1. Review of the Dirac Formula

In this section, we recall the construction of Dirac for the decomposition of the Hamiltonian vector field along a symplectic submanifold S⊂P, where (P,ω) is a symplectic manifold and *S* is symplectic, such that for all z∈S we have $T_{z}S⊕T_{z}S^{\omega }=T_{z}P$. Suppose that we are given a Hamiltonian function *H* on *P*. We will work semiglobally as follows. Suppose that *S* locally is expressed as the zero level set of a function $f:P\to R^{2k}$ that has 0 as a regular value. Then, we have dimS=2n−2k where dimP=2n and we denote $f=(f_{1},\ldots ,f_{2k})$. Since *S* is symplectic, we can restrict the Hamiltonian *H* to *S* and, pulling back the symplectic form to *S*, we have

$$\iota_{S}^{*}\omega \left(z\right)\left(X_{H|S}\left(z\right),v_{z}\right)=d\left(H|S\right)\left(z\right)\cdot v_{z}$$

for $v_{z}\in T_{z}S$.

**Proposition** **1.** *Each of the $X_{f_{i}}{|}_{S}$ is a section of $TS^{\omega }$. Furthermore, these sections are linearly independent and, at any point, span this distribution.*

**Proof.**  We have that $v\in T_{z}S$ if and only if $df_{i}\left(z\right)\cdot v=0$ for all i∈{1,…2k}. Now, let $v\in T_{z}S$. We then have, for each *i*, $\omega \left(z\right)\left(X_{f_{i}},v\right)=df_{i}\left(z\right)\cdot v=0$; therefore, $X_{f_{i}}{|}_{S}$ is a section of $TS^{\omega }$. From the independence of the $f_{i}$, a dimension count shows that the linearly independent $X_{f_{i}}\left(z\right)$ span $T_{z}S^{\omega }$, completing the proof. □

Now, define for each z∈P a (2k×2k) matrix by

$$C^{ij}\left(z\right)=\left\{f_{i},f_{j}\right\}\left(z\right).$$

We then have the following.

**Proposition** **2.** *Fix z∈S. The matrix $C^{ij}\left(z\right)$ is invertible.*

**Proof.**  Fix z∈S. We know that $T_{z}S$ is symplectic and, therefore, $T_{z}S^{\omega }$ is too. By the previous proposition, we know that the $X_{f_{i}}\left(z\right)$’s form a basis of the symplectic space $T_{z}S^{\omega }$. Therefore, the entry of the matrix $C^{ij}\left(z\right)$ is precisely the symplectic form evaluated on the vectors $X_{f_{i}}\left(z\right),X_{f_{j}}\left(z\right)$, which is, thus, a non-degenerate matrix since the restriction of ω to $T_{z}S^{\omega }$ is non-degenerate. □

We then have the following version of the Dirac formula for the Hamiltonian vector field.

**Theorem** **1.** *With the definition of $C^{ij}\left(z\right)$ given as above, denoting its inverse by $C_{ij}\left(z\right)$ for z∈S, the following formula holds:*

(1) $$X_{H|S}\left(z\right)=X_{H}\left(z\right)-\sum_{i,j=1}^{2k}C_{ij}\left(z\right)\left\{H,f_{i}\right\}\left(z\right)X_{f_{j}}\left(z\right).$$

**Proof.**  Since *S* is symplectic, we have that for all z∈S, $T_{z}P=T_{z}S⊕T_{z}S^{\omega }$. We will show that the projection $\pi_{z}:T_{z}P\to T_{z}S$ relative to this decomposition is given by the right-hand side of (1). This is equivalent to showing that $I-\pi_{z}:T_{z}P\to T_{z}S^{\omega }$ is given by

(2) $$\left(I-\pi_{z}\right)X_{H}\left(z\right)=\sum_{i,j=1}^{2k}\left\{H,f_{i}\right\}\left(z\right)C_{ij}\left(z\right)X_{f_{j}}\left(z\right).$$

To establish this, first observe that the right-hand side lies in $T_{z}S^{\omega }$ by Proposition 1. Next, if $H=f_{\ell }$ where ℓ∈{1,…2k}, then the right-hand side of (2) is

$$\begin{array}{cc} \sum_{i,j=1}^{2k}C^{\ell i}\left(z\right)C_{ij}\left(z\right)X_{f_{j}}\left(z\right) & =\sum_{j=1}^{2k}\delta_{\ell j}\left(z\right)X_{f_{j}}\left(z\right)=X_{f_{\ell }}\left(z\right) \end{array}$$

which shows that the operator is the identity on the subspace $T_{z}S^{\omega }$. Next, suppose that $X_{H}\left(z\right)\in T_{z}S$. Then, we have $df_{i}\left(z\right)\cdot X_{H}\left(z\right)=0$ for all *i* and, therefore, the right-hand side of (2) vanishes, as required. This proves that the projection $I-\pi_{z}$ is given by the Formula (2) and, thus, $\pi_{z}$ is given by the right-hand side of (1). □

Our main use of this theorem is to extend a Hamiltonian system on *S* to all of *P*. That is, given a Hamiltonian function *H* defined on *P*, we compute the right-hand side of Equation (1) for arbitrary z∈P to obtain a vector field defined on a neighborhood of the submanifold *S* (or level surface in this case) that will automatically be equal to $X_{H|S}$ on *S* itself. Thus, the Dirac formula gives us a way to extend Hamiltonian vector fields on *S* to the ambient space.

### 2.2. Feedback Integrators for Mechanical Systems with Holonomic Constraints

Given a Hamiltonian function *H* on a 2n-dimensional symplectic manifold *P* and its Hamiltonian vector field $X_{H}$ on *P*, consider a Hamiltonian system $\Sigma_{S}$ on a (2n−2k)-dimensional symplectic submanifold *S* of *P* whose Hamiltonian function is the restriction H|S of *H* to *S*. So, the equations of motion of $\Sigma_{S}$ can be written as

(3) $$\dot{z}=X_{H|S}\left(z\right),\;z\in S.$$

Assume that *S* is expressed as a level of a function $f=\left(f_{1},\ldots ,f_{2k}\right):P\to R^{2k}$. Thanks to the Dirac Formula (1), the dynamical system (3) extends to *P* as

(4) $$\dot{x}=X_{H}\left(x\right)-\sum_{i,j=1}^{2k}C_{ij}\left(x\right)\left\{H,f_{i}\right\}\left(x\right)X_{f_{j}}\left(x\right),\;x\in P.$$

It is easy to verify that *S* is an invariant manifold of (4). We now wish to integrate the dynamics (4) from a point $x_{0}$ in *S*.

Suppose that we can embed the manifold *P* in some Euclidean space $R^{m}$ with m≥2n and extend the vector field (4) to $R^{m}$, not necessarily in the Dirac way. Denote the extended vector field by *X* and write the corresponding dynamical system as

(5) $$\dot{x}=X\left(x\right),\;x\in R^{m}.$$

Suppose that both functions *f* and *H* also extend to the ambient Euclidean space $R^{m}$ in such a way that Df·X=0 and ∇H·X=0 in $R^{m}$, and that the manifold *S* is still a level set of the extension of *f* in $R^{m}$. As a result, the functions *f* and *H* are first integrals of (5) and the manifold *S* is an invariant manifold of (5).

Suppose that there are *ℓ* first integrals $I=(I_{1},\ldots ,I_{\ell })$ of (5) other than *H* and *f*, where the function *I* may include part of a function on $R^{m}$ whose level set defines *P* in $R^{m}$. Define a function $F:R^{m}\to R^{2k+1+\ell }$ by F=(f,H,I), and a function *V* on $R^{m}$ by

$$V\left(x\right)=\frac{1}{2}\left(F\left(x\right)-F\left(x_{0}\right)\right)K\left(F\left(x\right)-F\left(x_{0}\right)\right)$$

where $K=K^{T}$ is a (2k+1+ℓ)×(2k+1+ℓ) constant positive definite symmetric matrix. The minimum value of *V* is 0 and the function *V* satisfies

$$V^{-1}\left(0\right)=\left\{x\in R^{m}|x\in S,H\left(x\right)=H\left(x_{0}\right),I\left(x\right)=I\left(x_{0}\right)\right\}.$$

The set $V^{-1}\left(0\right)$ is an invariant manifold of (5) since $\nabla V\cdot X={\left(F\left(x\right)-F\left(x_{0}\right)\right)}^{T}K\cdot DF\cdot X={\left(F\left(x\right)-F\left(x_{0}\right)\right)}^{T}K\cdot 0=0$.

Modify the dynamical system (5) as follows:

(6) $$\dot{x}=X\left(x\right)-L\left(x\right)\nabla V\left(x\right),\;x\in R^{m},$$

where *L* is a map from $R^{m}$ into the set of m×m positive definite symmetric matrices and ∇V is computed as $\nabla V\left(x\right)=DF{\left(x\right)}^{T}K\left(F\left(x\right)-F\left(x_{0}\right)\right)$. Since 0 is the minimum value of *V*, the gradient ∇V vanishes on $V^{-1}\left(0\right)$, which implies that the two dynamical systems (5) and (6) coincide on $V^{-1}\left(0\right)$. It follows that the set $V^{-1}\left(0\right)$ is an invariant manifold of (6). Along any trajectory of (6), V(t) is an nonincreasing function of *t* since $\frac{dV}{dt}=\langle \nabla V,\left(X-L\nabla V\right)\rangle =-\langle \nabla V,L\nabla V\rangle \leq 0$; so, the trajectory converges to $V^{-1}\left(0\right)$ as t→∞ or it remains close to $V^{-1}\left(0\right)$ if it starts near $V^{-1}\left(0\right)$. Under some conditions on *V*, the set $V^{-1}\left(0\right)$ becomes a unique attractor of (6) in a neighborhood of itself in $R^{m}$; refer to [1] for those conditions. Due to the invariance of $V^{-1}\left(0\right)$ and the coincidence of (5) and (6) on $V^{-1}\left(0\right)$, integrating (5) from $x_{0}$ and integrating (6) from $x_{0}$ produce the same trajectory. Numerically, however, integrating (6) has the following advantage: if the trajectory deviates from $V^{-1}\left(0\right)$ at some numerical integration step, then it will get pushed back toward the attractor $V^{-1}\left(0\right)$, thus remaining on the manifold *S* and preserving all the first integrals; refer to [1,23] for a rigorous explanation. Although [1,23] provides some sufficient conditions for $V^{-1}\left(0\right)$ to be a local attractor of (6), in practice, it is not necessary to verify them. The procedure outlined in this section is good enough for applications, which will be illustrated with the planar and spherical pendulum in the next sections.

### 2.3. The Spherical Pendulum

We build a feedback integrator for the spherical pendulum [24], which is a Hamiltonian system with a holonomic constraint, and compare its performance with that of such geometric numerical integration methods as the RATTLE method, the Lie–Trotter splitting method, and the Strang splitting method. The phase space of the spherical pendulum is $T^{*}S^{2}$, which is a symplectic submanifold of $T^{*}R^{3}=R^{3}\times R^{3}$. This submanifold is globally defined as the $\left(\ell^{2},0\right)$-level set of the function $f=\left(f_{1},f_{2}\right):T^{*}R^{3}\to R^{2}$ with ℓ>0, where $f_{1}\left(q,p\right)={\left||q|\right|}^{2}$ and $f_{2}\left(q,p\right)=q\cdot p$ for $\left(q,p\right)\in T^{*}R^{3}$. We fix the Hamiltonian $H\left(q,p\right)={\left||p|\right|}^{2}/2m+mgq_{3}$. Restricted to $T^{*}S^{2}$, this gives the Hamiltonian of the spherical pendulum under gravity, with its $S^{1}$ symmetry. In order to write down the extended Hamiltonian vector field, we compute the following:

$$\begin{array}{cc} \left\{H,f_{1}\right\} & =-2f_{2}/m,\;\left\{H,f_{2}\right\}=-{∥p∥}^{2}/m+mgq_{3},\;\left\{f_{1},f_{2}\right\}=2f_{1},\\ X_{H} & =\left(p/m,-mge_{3}\right),\;X_{f_{1}}=\left(0,-2q\right),\;X_{f_{2}}=\left(q,-p\right), \end{array}$$

and

$$\left[C_{ij}\right]=\left[\begin{array}{cc} 0 & -1/(2f_{1})\\ 1/(2f_{1}) & 0 \end{array}\right],$$

where $e_{3}=\left(0,0,1\right)$. Hence, by the Dirac formula, the spherical pendulum system extends to $T^{*}R^{3}$ as

$$\dot{x}=X\left(x\right)$$

where

$$X\left(x\right)=\left(\frac{1}{m}p-\frac{f_{2}}{mf_{1}}q,-mge_{3}+\frac{f_{2}}{mf_{1}}p+\left(-\frac{{∥p∥}^{2}}{m}+mgq_{3}\right)\frac{1}{f_{1}}q\right)$$

for $x=\left(q,p\right)\in T^{*}R^{3}$. The extended system has four first integrals on $T^{*}R^{3}$: the constraint functions $f_{1}$ and $f_{2}$, the Hamiltonian *H*, and the vertical component $J\left(q,p\right)=q_{1}p_{2}-q_{2}p_{1}$ of angular momentum.

Choose a point $\left(q_{0},p_{0}\right)\in T^{*}S^{2}$, and define a function $V:T^{*}R^{3}\to R$ by

$$V\left(q,p\right)=\frac{1}{2}k_{1}|\Delta f_{1}{|}^{2}+\frac{1}{2}k_{2}|\Delta f_{2}{|}^{2}+\frac{1}{2}k_{3}{\left|\Delta H\right|}^{2}+\frac{1}{2}k_{4}{\left|\Delta J\right|}^{2},$$

where $k_{i}>0$, i=1,…,4 are constants; $\Delta f_{i}=f_{i}\left(q,p\right)-f_{i}\left(q_{0},p_{0}\right)$, i=1,2; $\Delta H=H\left(q,p\right)-H\left(q_{0},p_{0}\right)$; and $\Delta J=J\left(q,p\right)-J\left(q_{0},p_{0}\right)$. It is easy to verify that 0 is the minimum value of *V* and

$$V^{-1}\left(0\right)=\left\{\left(q,p\right)\in T^{*}R^{3}|f_{1}\left(q,p\right)=\ell^{2},f_{2}\left(q,p\right)=0,H\left(q,p\right)=H_{0},J\left(q,p\right)=J_{0}\right\},$$

where $H_{0}=H\left(q_{0},p_{0}\right)$ and $J_{0}=J\left(q_{0},p_{0}\right)$. The gradient of *V* is computed as

$$\nabla V=k_{1}\Delta f_{1}\nabla f_{1}+k_{2}\Delta f_{2}\nabla f_{2}+k_{3}\Delta H\nabla H+k_{4}\Delta J\nabla J,$$

where $\nabla f_{1}=\left(2q,0\right)$, $\nabla f_{2}=\left(p,q\right)$, $\nabla H=(mge_{3},p/m)$, and $\nabla J=(p\times e_{3},e_{3}\times q)$. Then, the feedback integrator corresponding to the function *V* for the spherical pendulum is given by

$$\dot{x}=X\left(x\right)-\nabla V\left(x\right)$$

or

(7) $$\begin{array}{cc} \dot{q} & =\frac{1}{m}p-\frac{f_{2}}{mf_{1}}q-2k_{1}\Delta f_{1}q-k_{2}\Delta f_{2}p-k_{3}mg\Delta He_{3}-k_{4}\Delta J\left(p\times e_{3}\right) \end{array}$$

(8) $$\begin{array}{cc} \dot{p} & =-mge_{3}+\frac{f_{2}}{mf_{1}}p+\left(-\frac{{∥p∥}^{2}}{m}+mgq_{3}\right)\frac{1}{f_{1}}q-k_{2}\Delta f_{2}q-k_{3}\frac{\Delta H}{m}p-k_{4}\Delta J\left(e_{3}\times q\right). \end{array}$$

We now compare the feedback integrator with the RATTLE method, the Lie–Trotter method, and the Strang method on the spherical pendulum system. The RATTLE algorithm is given on p. 246 in [5] and is known to be symplectic and convergent of order two [5]. The Lie–Trotter method and the Strang method are so-called splitting methods and are explained on pp. 253–254 in [5], where the two splitting methods yield first- and second-order numerical integrators, respectively [5]. For the splitting methods, we split the Hamiltonian function *H* into the kinetic function $H^{\left[1\right]}\left(q,p\right)={\left||p|\right|}^{2}/2m$ and the potential function $H^{\left[2\right]}\left(q,p\right)=mgq_{3}$, and we note that the dynamics associated to $H^{\left[1\right]}$ and $H^{\left[2\right]}$ can be integrated analytically. For numerical simulation, choose the parameter values m=g=ℓ=1 for convenience, and the initial points q(0)=(0,1,0) and p(0)=(1,0,−1) on $T^{*}S^{2}$. The corresponding initial values of the first integrals $f_{1}$, $f_{2}$, *H*, and *J* are $f_{10}=1$, $f_{20}=0$, $H_{0}=2$, and $J_{0}=-1$, respectively. We fix the time step size $h=10^{-3}$ and the time interval [0,100] for integration by all four methods. For the feedback integrator, the usual Euler scheme is used to integrate (7) and (8) with the following gain values: $k_{1}=50$, $k_{2}=50$, $k_{3}=50$, and $k_{4}=50$.

**Figure 1:**
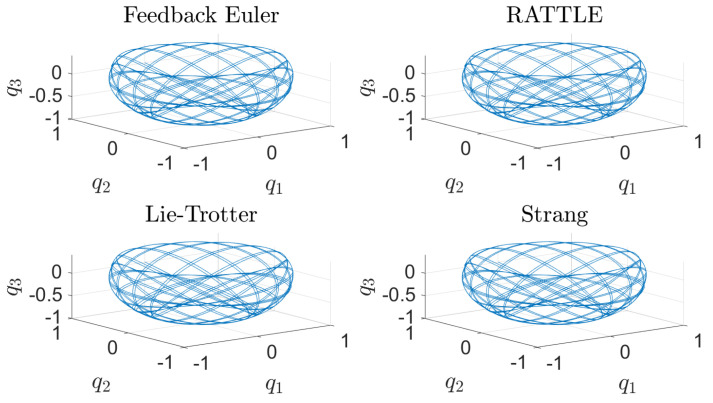
The trajectories of the position $q\left(t\right)=(q_{1}\left(t\right),q_{2}\left(t\right),q_{3}\left(t\right))$, 0≤t≤100, of the spherical pendulum generated by four different methods with step size $h=10^{-3}$: a feedback integrator with the Euler scheme, the RATTLE method, the Lie–Trotter splitting method, and the Strang splitting method.

**Figure 2:**
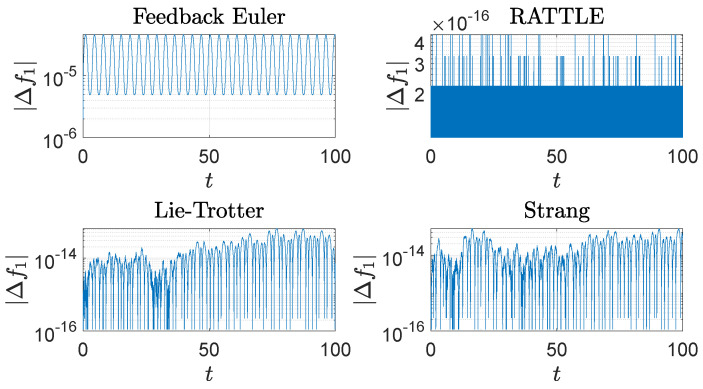
The trajectories of the deviation, $|\Delta f_{1}\left(t\right)|=|f_{1}\left(t\right)-f_{1}\left(0\right)|$, 0≤t≤100, of the spherical pendulum from the constraint set, ${∥q∥}^{2}=1$, generated by four different methods with step size $h=10^{-3}$: a feedback integrator with the Euler scheme, the RATTLE method, the Lie–Trotter splitting method, and the Strang splitting method. A logarithmic scale is used on the *y*-axis.

**Figure 3:**
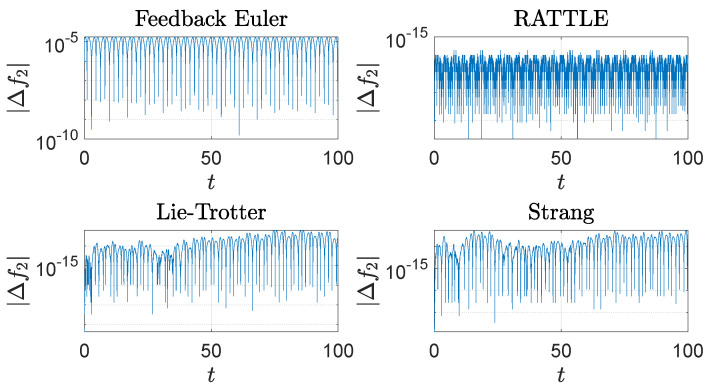
The trajectories of the deviation, $|\Delta f_{2}\left(t\right)|=|f_{2}\left(t\right)-f_{2}\left(0\right)|$, 0≤t≤100, of the spherical pendulum from the constraint set, q·p=0, generated by four different methods with step size $h=10^{-3}$: a feedback integrator with the Euler scheme, the RATTLE method, the Lie–Trotter splitting method, and the Strang splitting method. A logarithmic scale is used on the *y*-axis.

**Figure 4:**
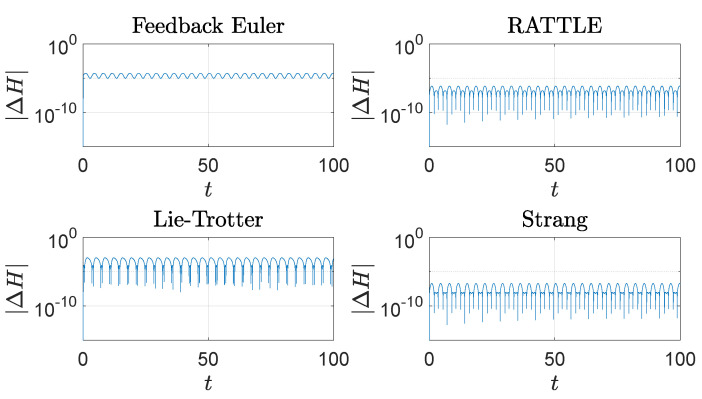
The trajectories of the energy error, |ΔH(t)|=|H(t)−H(0)|, 0≤t≤100, of the spherical pendulum generated by four different methods with step size $h=10^{-3}$: a feedback integrator with the Euler scheme, the RATTLE method, the Lie–Trotter splitting method, and the Strang splitting method. A logarithmic scale is used on the *y*-axis.

**Figure 5:**
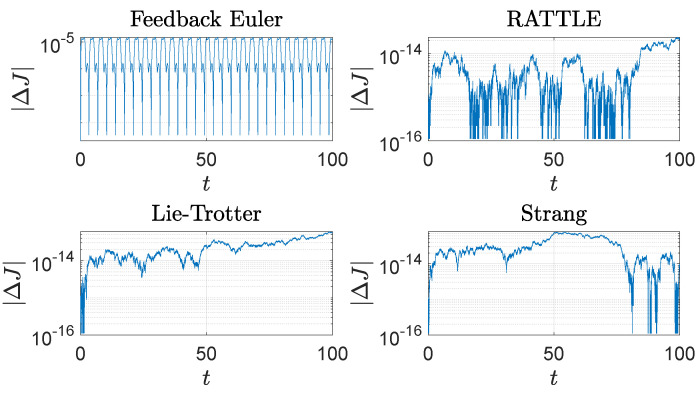
The trajectories of the momentum error, |ΔJ(t)|=|J(t)−J(0)|, 0≤t≤100, of the spherical pendulum generated by four different methods with step size $h=10^{-3}$: a feedback integrator with the Euler scheme, the RATTLE method, the Lie–Trotter method, and the Strang method. A logarithmic scale is used on the *y*-axis.

The computational results are plotted in Figure 1, Figure 2, Figure 3, Figure 4 and Figure 5. In Figure 1, the trajectories $q\left(t\right)=(q_{1}\left(t\right),q_{2}\left(t\right),q_{3}\left(t\right))$ of the configuration variables generated by the four methods are plotted. The feedback integrator with the Euler, RATTLE, Lie–Trotter, and Strang splitting methods all generate similar trajectories. Figure 2 and Figure 3 show the plots of the deviations $|\Delta f_{1}\left(t\right)|$ and $|\Delta f_{2}\left(t\right)|$ from the constraint manifold $T^{*}S^{2}$. The result by the RATTLE method is the best, and the trajectory produced by the feedback integrator with Euler remains close to $T^{*}S^{2}$ with the step size $h=10^{-3}$ taken into account. Likewise, the trajectories by the Lie–Trotter method and the Strang method stay close to $T^{*}S^{2}$, because the flows of the split Hamiltonians $H^{\left[1\right]}$ and $H^{\left[2\right]}$ each preserve $T^{*}S^{2}$, as does their composition.

The feedback integrator with Euler and the RATTLE method perform well in preservation of the values of the Hamiltonian *H* as do the other two methods, as shown in Figure 4. The vertical component *J* of angular momentum is well-preserved by all four methods as shown in Figure 5, where it is noticeable that the Lie–Trotter method and the Strang method perform very well in preservation of *J*.

The computational results imply that the feedback integrator with the first-order Euler scheme performs well on the spherical pendulum system in comparison with the well-known RATTLE method that is of second order, and to the Lie–Trotter method and the Strang method. An advantage of the feedback integrator over the other three methods is that it does not require any projection or solving of algebraic equations to stay on the holonomic constraint manifold. Further, it does not require any special integration algorithms, and simply employs well-known integration algorithms available such as Euler, Runge–Kutta, or Matlab ode45. Moreover, unlike the Lie–Trotter and Strang methods, which require a particular splitting of the dynamics into two parts that are separately integrable, the feedback integrator can be made to work for any constrained dynamical system by modifying the vector field as in (6).

### 2.4. The Simple Pendulum

One potential drawback of the feedback integration method is that the feedback vector field is more complex due to the presence of the stabilizing forces. For example, the right-hand side of (7) and (8) is a sum of five gradients (one gradient of the Hamiltonian and four gradients of the feedback potential) and this cost compounds for higher-order numerical methods, which typically require several force evaluations per integration step. This added computational cost must be taken into account when comparing the error profile of feedback integrators with that of standard methods, such as RATTLE, which only evaluate the gradient of the Hamiltonian (but possibly multiple times per integration step).

In this section, we show that the increase in complexity is compensated by the approximate conservation properties of the integrator, and in particular, we show that feedback integrators are at least as effective as standard integrators when the computational budget is taken into account. We compare the performance of feedback integrators of different orders with that of the RATTLE method and show that the increase in computational cost is balanced by the fact that feedback integrators require fewer force evaluations overall to achieve a given accuracy.

To assess the global error of the various integrators, we take recourse to a simpler mechanical system, for which exact solutions are known and can be approximated with arbitrary precision: the simple gravitational pendulum. The simple pendulum consists of a mass *m* that is free to swing at the end of a rigid rod of length *ℓ* under the influence of gravity. The motion of the pendulum takes place entirely in a fixed plane, denoting the angle between the horizontal and the position of the pendulum by θ, is determined by

(9) $$\ddot{\theta }+g\cos\theta =0,$$

where *g* is the gravitational acceleration. The dynamics of the pendulum as a constrained system can be derived directly via a calculation as in the previous section, or by observing that the spherical pendulum naturally moves in a fixed plane if the initial position, momentum, and direction of gravity are all coplanar. Either way, the extended Hamiltonian vector field is readily seen to be

$$X\left(x\right)=\left(\frac{1}{m}p-\frac{f_{2}}{mf_{1}}q,-mge_{y}+\frac{f_{2}}{mf_{1}}p+\left(-\frac{{∥p∥}^{2}}{m}+mgy\right)\frac{1}{f_{1}}q\right),$$

where q=(x,y) and $p=(p_{x},p_{y})$ are the coordinates and momenta of the pendulum, respectively, and $e_{y}=\left(0,1\right)$. The first integrals on $T^{*}R^{2}$ are the Hamiltonian $H\left(q,p\right)={∥p∥}^{2}/2m+mgy$ and the constraint functions

$$f_{1}\left(q,p\right)={∥q∥}^{2}=x^{2}+y^{2}\;\mathrm{and}\;f_{2}\left(q,p\right)=q\cdot p=xp_{x}+yp_{y}.$$

Similar to the spherical pendulum, we consider these three conserved quantities and for given initial values $\left(q_{0},p_{0}\right)\in T^{*}S^{2}$, we define the function $V:T^{*}R^{2}\to R$ given by

$$V\left(q,p\right)=\frac{1}{2}k_{1}|\Delta f_{1}{|}^{2}+\frac{1}{2}k_{2}|\Delta f_{2}{|}^{2}+\frac{1}{2}k_{3}{\left|\Delta H\right|}^{2},$$

where $k_{1},k_{2},k_{3}$ are positive constants; $\Delta f_{i}=f_{i}\left(q,p\right)-f_{i}\left(q_{0},p_{0}\right)$; i=1,2; and $\Delta H=H\left(q,p\right)-H\left(q_{0},p_{0}\right)$. The feedback integrator for the simple pendulum then becomes

(10) $$\begin{array}{cc} \dot{q} & =\frac{1}{m}p-\frac{f_{2}}{mf_{1}}q-2k_{1}\Delta f_{1}q-k_{2}\Delta f_{2}p-k_{3}mg\Delta He_{2} \end{array}$$

(11) $$\begin{array}{cc} \dot{p} & =-mge_{2}+\frac{f_{2}}{mf_{1}}p+\left(-\frac{{∥p∥}^{2}}{m}+mgy\right)\frac{1}{f_{1}}q-k_{2}\Delta f_{2}q-k_{3}\frac{\Delta H}{m}p. \end{array}$$

While the pendulum can, in principle, be integrated exactly, obtaining the solution as a function of time is not straightforward and requires inverting the elliptic integral of the first kind. To avoid this difficulty, we integrate the Equation (9) for θ using a high-order Runge–Kutta method with tolerance set to $10^{-13}$. For all numerical simulations, we set m=g=ℓ=1, and we use q(0)=(1,0) and p(0)=(0,0) as the initial conditions. For the feedback method, we use three off-the-shelf numerical schemes to integrate (10) and (11): (a) the forward Euler method, (b) the explicit 4th-order Runge–Kutta method (RK4), and (c) the Dormand–Prince method of 8th order (DOP853). Note that the Dormand–Prince method is a variable step-size integrator while all the others use a fixed step size.

**Figure 6:**
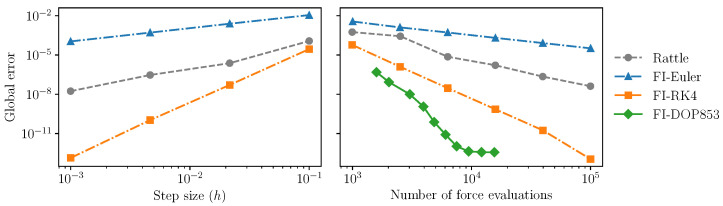
Left: The global trajectory error after one period of the pendulum (approximately 7.4 time units) as a function of the step size (DOP853 is not included as it is a variable step size method). As the step size decreases, the global error decreases at a rate proportional to the error of the method. Right: The global error, but now as a function of the computational budget (number of force evaluations). Larger computational budgets correspond to smaller step sizes and, hence, lower errors, but take into account the fact that the feedback methods involve more force evaluations. Despite the overhead, feedback integrators are able to do at least as well as, or better than, RATTLE.

Figure 6 (left) shows the trajectory error after one period of the pendulum between the standard RATTLE integrator on the one hand, and the three feedback integrators on the other hand, as a function of the step size. The global error decreases as the step size decreases, in line with the order of the method. This is to be expected, since the feedback approach merely modifies the vector field to be integrated, but does not otherwise alter the underlying numerical method.

The vector field integrated by the feedback methods is more complex; thus, one can ask what the impact is on the total execution time. To investigate this question, we give each method a fixed computational budget and modify the integrator code so that each evaluation of the vector field reduces the computational budget by a certain amount. For evaluations of the feedback vector field (10) and (11) the cost per evaluation is set to 4, since the vector field consists of 4 gradients, whereas for the evaluation of the gradient of the Hamiltonian (needed e.g., by RATTLE), the cost per evaluation is 1. We then compare the performance of the integrators over one period of the pendulum and adjust the step size (for RATTLE and the feedback Euler and RK4 methods) or the tolerance (for the feedback-DOP853 method, which uses a variable step size) so that the computational budget is exhausted over one period.

The result is shown in Figure 6 (right), which shows the global error as a function of the computational budget. Note that the error for the feedback-DOP853 method stabilizes somewhat below $10^{-12}$. This is roughly the point where we encounter the limits in the accuracy of the exact trajectories (which were obtained by numerical integration of (9), where the tolerance was set to $10^{-13}$).

We see that feedback integrators are able to integrate the dynamics of the underlying system accurately (i.e., with low error) and efficiently (using comparable or lower numbers of force evaluations), compared with specific holonomic integrators. In terms of computational efficiency, higher-order integrators such as the feedback-DOP853 integrator clearly achieve better results than others (lower-order feedback methods and RATTLE). This again demonstrates one of the key benefits of the feedback integrator method, showing that it is possible to use any standard numerical integration scheme to obtain approximate constraint preservation, without loss of accuracy or computational efficacy.

## 3. Conclusions

We have presented a general framework to extend the feedback integrators [1] to systems with holonomic constraints. Beginning with a symplectic submanifold *S* in the symplectic manifold *P*, where *S*—the holonomic constraint—is the level set of $f_{1}\ldots f_{2k}$, on which we have a Hamiltonian H|S. We use the Dirac formula to extend the vector field $X_{H|S}$ to a vector field *X* on *P*. We then apply the feedback integrator to *X* using an embedding of *P* in Euclidean space.

More specifically, in the case that the symplectic manifold *S* is of the form $T^{*}Q$, where *Q* embeds in $R^{n}$ and $T^{*}Q$ embeds in $T^{*}R^{n}$ as the level set of functions $f_{1},\ldots f_{2k}$, on which we have an extended Hamiltonian *H*, we can compute the extension of the vector field $X_{H|T^{*}Q}$ directly from the Dirac formula (1). With this vector field, now defined on a Euclidean space, $T^{*}R^{n}$, we construct the function $V:T^{*}R^{n}\to R_{\geq 0}$, whose 0 level set contains the dynamic invariants for a given initial point in *S*, where the set of dynamic invariants includes the functions $f_{1},\ldots f_{2k}$. The feedback-integrator-modified vector field, constructed from the extended vector field *X* by adding the negative gradient of *V*, is fed into *any* integrator, for example, first-order Euler. In addition to the dynamic invariants, the integrator automatically respects, from the construction of the modified vector field, the holonomic constraints. The resulting integrator has superior performance to even symplectic integrators, which depend, typically, on implicit solvers. As future work, we plan to examine the problem of optimal control on manifolds and its relationship with Hamiltonian mechanics from the viewpoint of feedback integrators about which some preliminary works have been carried out in [25,26,27]. We also plan to examine the effect of the magnitude of feedback integrator term on the precision of numerical integration and to extend feedback integrators to field theory.

## Data Availability

Not applicable.
